# Laparoscopy in pancreatic tumors

**DOI:** 10.4103/0972-9941.33272

**Published:** 2007

**Authors:** S V Shrikhande, S G Barreto, P J Shukla

**Affiliations:** Department of Gastrointestinal and Hepatopancreatobiliary Surgical Oncology, Tata Memorial Hospital, Mumbai - 400012, India

**Keywords:** Laparoscopy, pancreas, tumors

## Abstract

Recently, increasing number of manuscripts - original articles and case reports have attempted to provide evidence of the forays of minimal access surgery into pancreatic diseases. Many, based on the lack of Level I evidence, still believe that laparoscopy in pancreatic surgery is experimental. This article attempts to look into data exploring the existing use of minimally invasive surgery in pancreatic disease to answer a vital question - what does the evidence say on the current status of laparoscopic surgery in pancreatic tumors.

## INTRODUCTION

Gagner and Pomp[1] have been credited with the first major laparoscopic attempt in pancreatic surgery. While, they performed what was believed to be an almost impossible task - laparoscopic pylorus preserving pancreaticoduodenectomy, the association between laparoscopy and the pancreas dates back to as early as 1911. Berheim, Meyer-Burg, Cuschieri and Ishida[2–5] had explored the role of laparoscopy as a staging tool for pancreatic diseases. The slow progress of laparoscopy in pancreatic surgery has been attributed to the retroperitoneal location of the gland, its proximity to various important vascular structures and organs, especially the superior mesenteric vessels, splenic vessels and the spleen, as well as the friable nature of the gland and the difficulty in its exposure.[6] Today, laparoscopy has been used as a diagnostic and therapeutic tool in the management of pancreatic diseases. The prime indications for the role of laparoscopy in pancreatic diseases include: 1) staging of pancreatic cancer, 2) resection of benign and malignant diseases, 3) drainage procedures in pancreatitis and pseudocysts and finally, 4) in the palliation and pain relief in pancreatic cancer.

## STAGING OF PANCREATIC CANCER

The ability of laparoscopy to detect occult liver and peritoneal metastasis led to it being touted as a vital preoperative test for evaluation. It has also been used to obtain peritoneal fluid for cytology following instillation of saline into the peritoneal cavity.[7] However, as the sensitivity of computed tomography (CT) increased, especially so following the introduction of multi detector CT scans (MDCT) and standard protocols for reporting of these investigations,[8,9] the indications for diagnostic laparoscopy for staging of pancreatic cancer began to reduce. The role of laparoscopy in this setting was downplayed by the fact that it was unable to detect locally advanced lesions due to its permissible two-dimensional inspection of the liver and peritoneal surfaces. Also, lack of tactile sensation precludes detection of intraparenchymal liver disease.

In the light of these findings, laparoscopy for staging of pancreatic cancers improved only after the introduction of laparoscopic ultrasonography. This not only enabled the detection of locally advanced disease and intraparenchymal liver involvement, but also gave a good indication of vascular involvement.[10]

Pisters *et al.*[11] in a thorough review of the available literature on laparoscopy for detection of occult M1 disease have listed the following indications for staging laparoscopy:

1. Larger primary tumors
2. Lesions in the neck, body or tail of the pancreas
3. Equivocal radiographic findings suggestive of occult M1 disease, such as low volume ascites, CT findings indicating possible carcinomatosis and small hypodense regions in the hepatic parenchyma that suggest hepatic metastases that are not amenable to percutaneos biopsy and
4. Subtle clinical and laboratory findings suggesting more advanced disease (e.g., marked hypoalbuminemia and / or weight loss, significant increase in CA 19-9 levels and relatively severe pain requiring narcotic analgesia)

Such patients should be considered for laparoscopic staging performed either as a separate procedure or before the planned pancreatic resection. They concluded that laparoscopy may detect metastasis in 4-15% patients with no evidence of metastases on contrast enhanced CT scans.

## LAPAROSCOPY FOR PALLIATION OF PANCREATIC CANCER

Today, endoscopic biliary stenting continues to be the most widely performed procedure for palliation in patients with malignant biliary obstruction secondary to pancreatic cancer. Van den Bosch *et al.*,[12] however felt that such a form of palliation was fraught with problems of stent blockade with the need for restenting especially in female patients who survived due to inherently small tumors, without liver metastases.

In randomized trials, endoscopy has failed to show any benefits with respect to biliary decompression and overall survival, over surgical palliation.[13–15] The reason being that while the immediate benefits of shorter hospital stay are better in the endoscopy arm, these benefits are offset by the increased risk of obstruction in the patients with endobiliary prosthesis when patients survive for longer durations.

The main two forms of surgical palliation of malignant obstruction in pancreatic cancer are choledochoenterostomy (CDE) and cholecystoenterostomy (CCE). With regard to outcomes (complications and bypass failure), CDE has been shown to be better than CCE.[16] While both these procedures have been described laparoscopically, technically, CCE is easier to perform.[17]

It has been suggested that when a patient is explored for a pancreaticoduodenectomy and found inoperable, a prophylactic retrocolic gastrojejunostomy should be added to a biliary bypass to overcome late gastric outlet obstruction.[18,19] Laproscopic gastroenterosotomy can now be performed laparoscopically with satisfactory outcomes.[20,21]

However, laparoscopic palliation of locally advanced pancreatic cancer demands a level of technical expertise that can be found only in specialized centers. Hence, the overall applicability of these techniques to patients with unresectable pancreatic disease treated by surgeons not practicing at specialized centers remains unknown. Randomized studies are necessary to determine the general applicability of laparoscopic biliary and gastric diversion in the treatment of locally advanced pancreatic cancer.[22]

With a recurrence of pain noted approximately two to five years following nerve ablation, bilateral thoracoscopic splanchnicectomy is a very useful method for palliation of intractable pain in pancreatic cancer. It helps reduce the dosage of opiates required and provides a better quality of life in these terminal patients.[23]

## LAPAROSCOPY FOR BENIGN PANCREATIC TUMORS

Benign tumors of the pancreas, especially endocrine tumors, like insulinomas and cystadenomas, that are predominantly located in the distal body and tail of the pancreas, are ideally suited for the laparoscopic approach. This is because the specimen is small and contrasts greatly with the size of the abdominal incision required to access this retroperitoneal gland at conventional open surgery; also there is no need to fashion an anastomosis.[24] With over 400 reported cases in English literature,[25] laparoscopic distal pancreatectomy (LDP) and enucleation have not only been shown to be feasible, but in experienced hands, it has been associated with shorter operating times, early return of bowel function, minimal narcotic requirements and early resumption of normal activities.[26,27] Table 1 highlights recent studies of LDP and their outcomes.[28–32] An important problem leading to conversion in most studies has been the difficulty in localizing the tumor using laparoscopy.

**Table 1:** Recent studies of laparoscopic distal pancreatectomy for pancreatic tumors highlighting their outcomes

Authors	Patient	Intervention	Conversion	Types of complications, numbers
Patterson, *et al*.[28]	19	Enucleation = 4	2 + 1 hand assisted	Fistulae = 3
		Pancreaticosplenectomy = 12		
		Spleen preserving distal pancreatectomies = 3		
Park, *et al*.[29]	25	Enucleation = 2		
		Pancreaticosplenectomy = 11	Enucleation = 1	Collections = 1
		Spleen preserving distal pancreatectomies = 12	Distal pancreas = 1	
			2 hand assisted	
Dulucq, *et al*.[30]	21	Pancreaticosplenectomy = 5	Distal pancreas = 1	Fistula = 1
		Spleen preserving distal pancreatectomies = 16		Abscesses = 2
				Haemorrhage = 1
				Eventration of port site = 1
Toniato, *et al*.[31]	12	Enucleation = 4	1	4
		Pancreaticosplenectomy = 2		
		Spleen preserving distal pancreatectomies = 5		
Edwin, *et al*.[32]	24	Enucleation = 7		
		Pancreaticosplenectomy = 12		
		Spleen preserving distal pancreatectomies = 5		
			4	9

Hand-assisted laparoscopic surgery (HALS) came into being with one of its prime benefits being the ability to maintain tactile sensation. HALS in pancreatic surgery is yet in its infancy.[33] While early reports have been encouraging, there are some who believe that the use of the hand causes an encroachment of the external workspace, hindering ideal positioning of the instrument ports[34] as well as an increased risk of wound related problems. Clearly, larger studies with long-term follow up are needed to define its true indications.

As regards complications, hemorrhage (necessitating intraoperative conversion), fistula formation, collections and wound related problems have been reported. To be able to actually compare the incidences of these complications with open surgery would be too premature considering that the largest study in literature for open conventional distal pancreatectomies is 235[35] cases as compared to 23 in case of LDP.

Fernandez-Cruz *et al.*,[36] have further performed laparoscopic enucleations on patients with sporadic and multiple insulinomas based on preoperative localization using endoscopic ultrasonography, CT scan and ^111^Octreoscan.

Sa Cunha *et al.*[37] have recently compared open and laparoscopic surgery for solitary insulinomas and have concluded that the safety, feasibility, lower incidence of fistulae and reduced hospital stay indicate that this procedure is definitely emerging as an effective method of managing small, well localized tumors of the body and tail.

## LAPAROSCOPIC PANCREATICODUODENECTOMY

The feasibility of this procedure has been demonstrated by studies exploring this procedure for chronic pancreatitis, lower common bile duct cancer and ampullary tumors.[1,38–40] While majority of these reports are case reports, the largest series[30] is of ten patients (six patients were completed laparoscopically and four patients required a minilaparotomy for the reconstruction).

The realization is that while the resection is technically possible, the level of difficulty arises at the time of performance of the reconstruction. This has led to many surgeons performing the resection laparoscopically and then following it up by a mini-laparotomy for the reconstruction or performing the entire surgery as laparoscopic-assisted[41,42] or hand-assisted.[43–45]

Another problem with this procedure is the doubts raised as regards the level of oncological radicality that can be obtained especially when tissues along the superior mesenteric vessels and lymph nodes are not adequately removed.[29]

From the available literature, there appears to be no benefit of the laparoscopic approach over the conventional open approach.[29,46,47]

## CONCLUSION

Minimally invasive surgery, as a whole, does have many benefits. While we have realized the advantages of laparoscopy in some surgeries to the fullest (laparoscopic cholecystectomy and laparoscopic fundoplication), apart from selected patients undergoing distal pancreatectomy for small tumors, it is too premature to presume that laparoscopy can be used in tumors of the pancreas. In the case of pancreaticoduodenectomy, it seems that laparoscopy offers no advantage over conventional pancreaticoduodenectomy. This is perhaps because the morbidity of the procedure does not lie within the wound, but rather, it lies in what is done inside the abdomen. It is a procedure that should be performed only by expert laparoscopists who should appropriately select patients and tumors. We have sufficient evidence in open surgery that large volumes do help in improving outcomes of complex procedures. It thus appears logical to extrapolate this experience to laparoscopic pancreatic surgery. What is certain is that high volume centers will have to take a proactive role in facilitating training in laparoscopic pancreatic surgery. It is only when we approach the path ahead in such a systematic manner that we will be able to conduct the required randomized trials necessary to obtain sufficient data on long term outcomes. At this point it is pertinent to note that feasibility should not be confused with benefit.
